# Immune Boosting Explains Regime-Shifts in Prevaccine-Era Pertussis Dynamics

**DOI:** 10.1371/journal.pone.0072086

**Published:** 2013-08-26

**Authors:** Jennie S. Lavine, Aaron A. King, Viggo Andreasen, Ottar N. Bjørnstad

**Affiliations:** 1 Department of Ecology and Evolutionary Biology, University of Michigan, Ann Arbor, Michigan, United States of America; 2 Fogarty International Center, NIH, Bethesdsa, Maryland, United States of America; 3 Department of Mathematics, University of Michigan, Ann Arbor, Michigan, United States of America; 4 Center for the Study of Complex Systems, University of Michigan, Ann Arbor, Michigan, United States of America; 5 Department of Science, Systems and Models, Roskilde University, Roskilde, Denmark; 6 Department of Entomology and Biology, Pennsylvania State University, University Park, Pennsylvania, United States of America; Albert Einstein College of Medicine, United States of America

## Abstract

Understanding the biological mechanisms underlying episodic outbreaks of infectious diseases is one of mathematical epidemiology’s major goals. Historic records are an invaluable source of information in this enterprise. Pertussis (whooping cough) is a re-emerging infection whose intermittent bouts of large multiannual epidemics interspersed between periods of smaller-amplitude cycles remain an enigma. It has been suggested that recent increases in pertussis incidence and shifts in the age-distribution of cases may be due to diminished natural immune boosting. Here we show that a model that incorporates this mechanism can account for a unique set of pre-vaccine-era data from Copenhagen. Under this model, immune boosting induces transient bursts of large amplitude outbreaks. In the face of mass vaccination, the boosting model predicts larger and more frequent outbreaks than do models with permanent or passively-waning immunity. Our results emphasize the importance of understanding the mechanisms responsible for maintaining immune memory for pertussis epidemiology.

## Introduction

**Figure 1:**
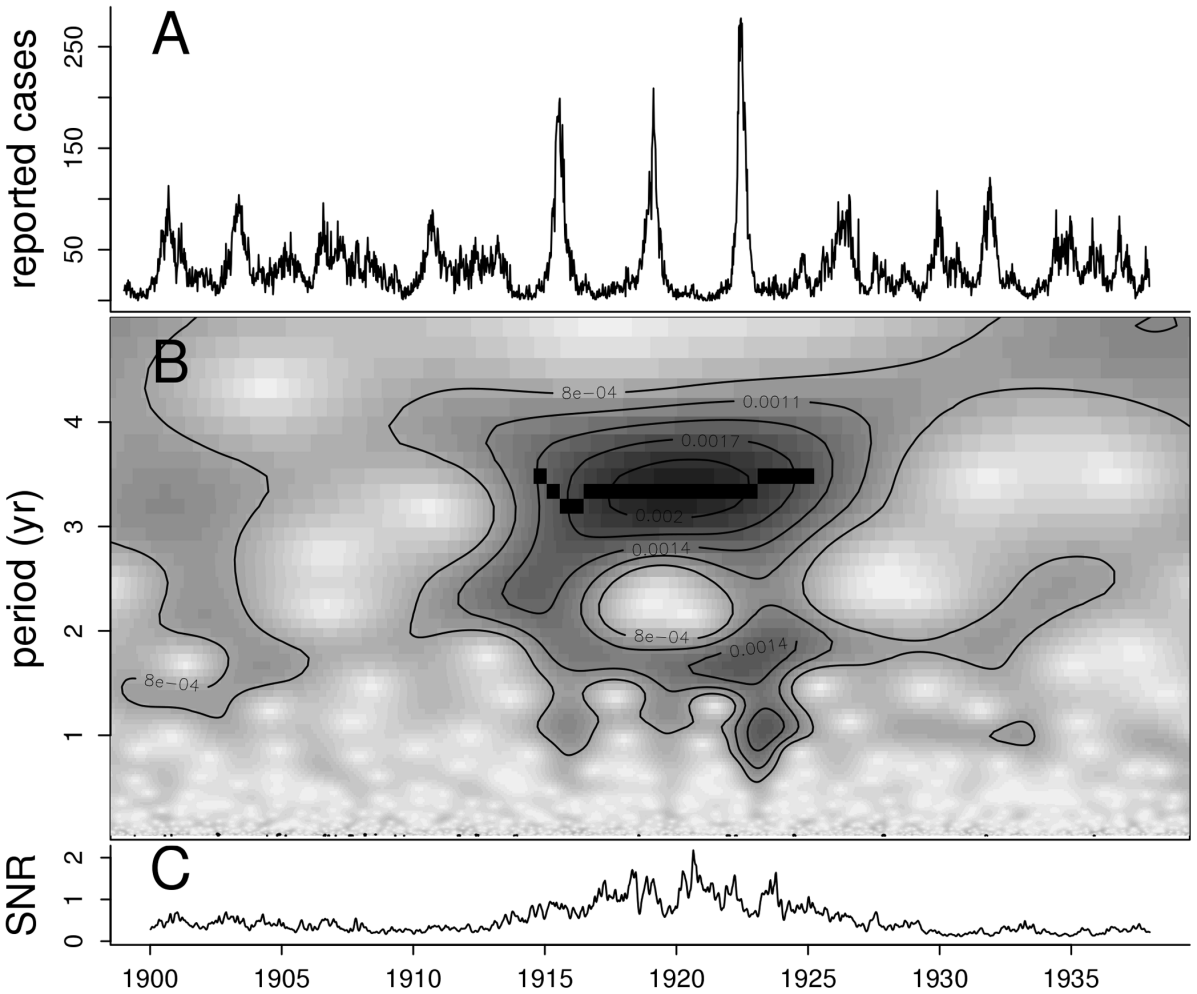
Time series of reported pertussis cases in Copenhagen (A), wavelet decomposition of the square-root transformed reports (B), and the signal-to-noise ratio (C). Darker areas of the wavelet plot indicate stronger support for cycles of the period identified on the middle left axis. The black band shows the region with strong cycles as identified by the ridge-finding algorithm.

Whooping cough (pertussis) outbreaks are highly variable in size and regularity. Pertussis time series from diverse countries, including Denmark, Algeria, Japan, and the United States, exhibit a peculiar kind of variability: intermittent bouts of large multiannual epidemics interspersed with periods of less pronounced cycles [1]. Herein we refer to this as “regime-switching” behavior. Changes in temporal patterns of disease incidence can often be attributed to secular changes in key demographic or climatic drivers [2], [3], but not in this case: though trends in birth rate and vaccine coverage have been shown to cause shifts in the *duration* of pertussis’ interepidemic period [4], no such trends are generally associated with the regime-switching behavior [1]. An age-specified, weekly time series of pertussis reports from Copenhagen in the early 20th century provides an excellent illustration of regime-switching, displaying distinct shifts between low-amplitude, noisy multiannual cycles and high-amplitude cycles marked by a high signal-to-noise ratio (Fig. 1). These data uniquely combine weekly incidence reports from a large city with detailed demographic data on births and population size in the prevaccine era. They allow us to glimpse a portrait of pertussis’ natural history before the vaccine was widely used. Vaccination affected not only immunity and transmission among vaccinated human populations, but also the genetic make-up of *Bordetella pertussis* populations [5]. Here, we examine the hypothesis that an interaction between transmission, waning immunity, and stochasticity underlies the regime-switching behavior.

Three biological mechanisms are thought to contribute to the cyclic nature of pertussis outbreaks. First, the long infectious period, estimated at between three and four weeks, renders the dynamics sensitive to stochastic perturbations, which in turn leads to transient multi-annual cyclicity [6]. Second, infections stimulate immunity; those who have been infected are typically protected against subsequent infection for several years at least, which in turn leads to nonlinear feedbacks in the dynamics. Third, components of the immune memory response can be stimulated by re-exposure, potentially resulting in prolongation of protection following infection [7]–[10] and the potential for positive feedbacks between incidence and population-level immunity. Historically, pertussis was thought of as a classic, permanently immunizing, childhood infection, with the mean age of infection around five years and few reports of re-infection [11], [12]. Recently, however, increasing incidence among teenagers in highly vaccinated populations has led doctors and scientists to reconsider the duration of pertussis immunity. It is now widely believed that vaccine-induced immunity wanes and that infections in older individuals contribute to transmission [13]–[17]. Moreover, cross-sectional serological surveys and clinical records suggest that infection-induced immunity may not be life-long either [7],[18],[19].

We have recently shown that deterministic models combining impermanent immunity with rapid immune boosting can capture the change in age-incidence following vaccination, and predict, under some circumstances, coexistence of cyclic and fixed-point attractors [20]. These models also predict an interesting interplay between the dynamic regime and the age-specific incidence in which a dynamic regime change to large-amplitude cycles with deep troughs drives increased incidence among older age-groups [20]. Two questions arise: (i) How do stochasticity and immune boosting together affect the amplitude and frequency of cyclic pertussis outbreaks? (ii) Can this model shed light on the apparent regime-switching in pertussis dynamics?

A detailed quantitative accounting of observed disease dynamics must be based on models that combine (i) secular trends in key parameters, such as birth rate, (ii) external periodic drivers, such as seasonality, and (iii) stochasticity intrinsic to the transmission process [21]. Here, we first explore the dynamical and age-specific implications of immune boosting and stochasticity in pertussis epidemiology and then attempt to distinguish among several possible explanations of the observed dynamical behavior of pertussis via model comparison. We fit stochastic models of pertussis transmission and immune dynamics to the time series of total disease incidence in prevaccine-era Copenhagen.

**Figure 2:**
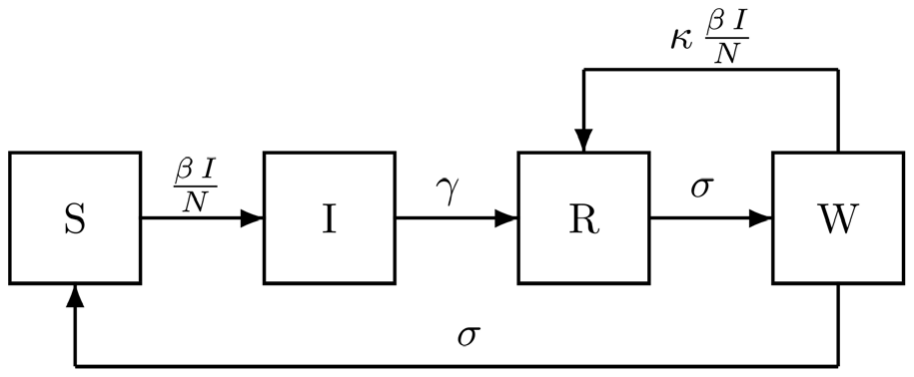
Flow diagram for the models, showing rates of flow among compartments containing individuals who are susceptible to pertussis (S), infected and infectious (I), recovered and immune (R), and susceptible to boosting because their immunity is waning (W). When 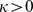 and 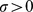, immunity wanes and is boosted by re-exposure; we term this the SIRWS model. When 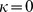 and 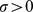, immunity wanes passively; we term this the SIRS model. In this model, the duration of immunity is gamma-distributed with mean 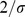 and variance 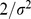. When 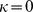 and 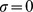, immunity is permanent; this is the SIR model.

We compare a nested set of models with different roles for immunity (Fig. 2) to these data. Under the first model (SIR), re-exposure never results in reinfection. In the second, immunity wanes passively and subsequent exposure can lead to infection (SIRS). In the third, waning immunity may be boosted by reexposure (SIRWS). All the models integrate concurrent data on births and population size and thus accomodate the possibility that the observed regime-switching is due to secular changes in these drivers. We compare the models using maximized likelihood and explore their consequences.

We show (i) the best model incorporates both immune waning and boosting, though we are near the limit of the data’s ability to distinguish between the models; (ii) the boosting model exhibits regime-switching behavior that can be related to two coexisting cyclic attractors created by the interplay between waning and boosting of immunity; and (iii) the two most viable models (SIRS and SIRWS) make distinct quantitative predictions in the presence of vaccination.

## Results

### Time-series Analysis

The data exhibit two types of dynamical behavior. Strong three-year cycles are present between 1914 and 1924, whereas the rest of the time series exhibits only weak signals of multiannual cyclicity (Fig. 1). The cycles between 1914 and 1924 are distinguished from the rest of the time series by: their large amplitude (Fig. 1A), their increased signal-to-noise ratio (Fig. 1C), and a ridge in the wavelet spectrum (black band in Fig. 1B). Neither birth rate, nor population growth show any conspicuous change preceding the entrance into the large cycles or the reversal back to the low-amplitude regime (Figs. S1 and S2 in File S1), and there is no other obvious external driver, such as changes in school attendance policies [22].

**Figure 3:**
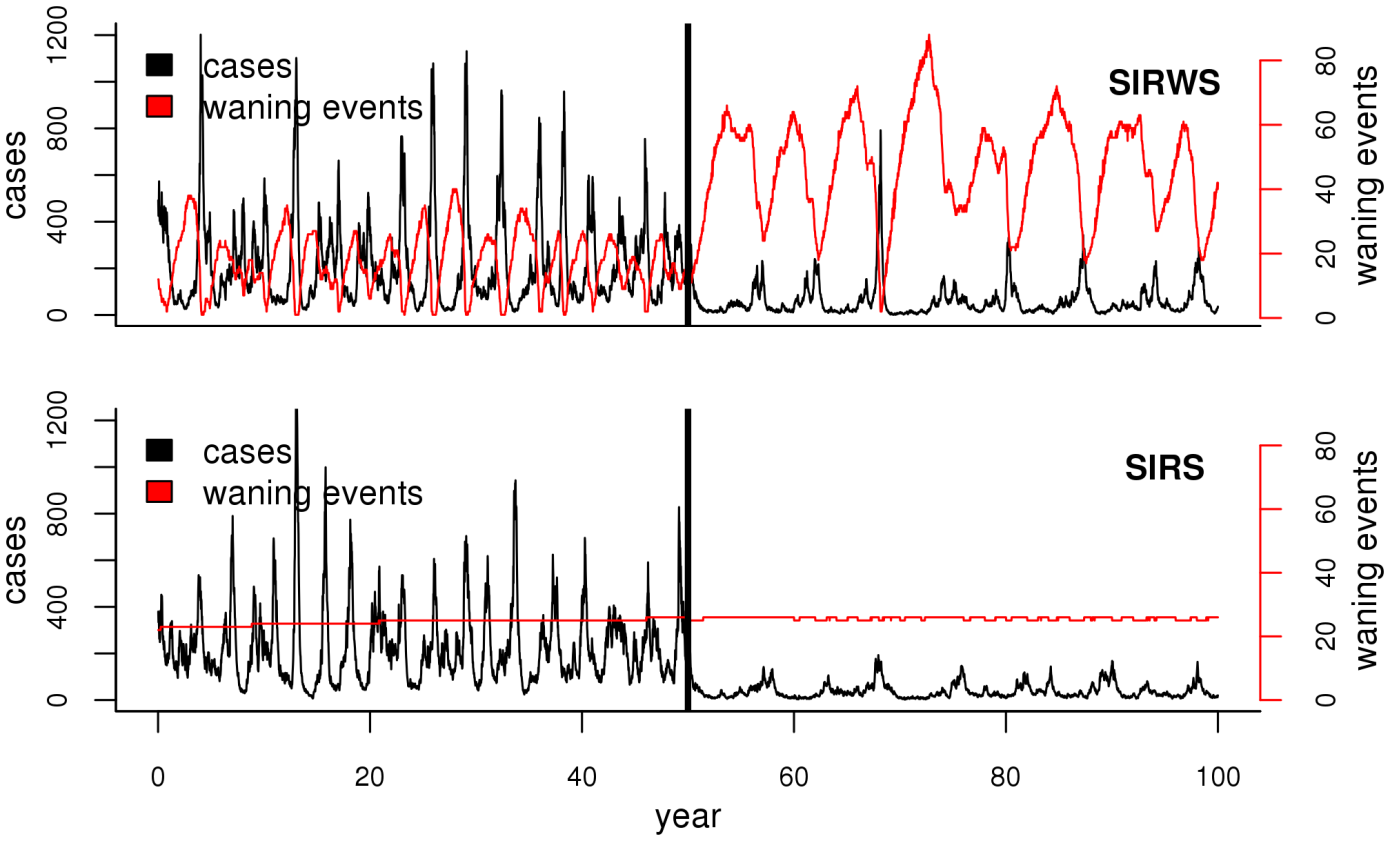
Sample simulations from the MLEs of the two best-supported models. The top panel shows the model with boosting (SIRWS), the bottom panel the model with passive waning of immunity (SIRS). In each, the black curve shows the weekly number of reported cases, the red curve the number of waning events that occurred each week. The thick vertical black line indicates the start of 90% vaccine coverage. The simulations have been carried out with the birth rate and population size fixed at their historical averages.

We estimated model parameters by maximum likelihood using iterated filtering [23]–[25]. We distinguished among the various models using likelihood ratio tests (LRT) and AIC weights [26]. The model with both waning and boosting (SIRWS) provided the best fit to the data (Table 1). The model with life-long immunity (SIR) received essentially no support. The model with waning but no boosting (SIRS) received marginal support. Under this model, a single exposure to pertussis induces immunity that on average is very long-lived. However, the model implies a wide variance in the durations of immunity, 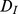, with 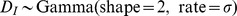. Therefore, although the rate of immune decay is small, the model predicts a substantial contribution of loss of immunity to susceptible recruitment (Fig. 3, bottom panel): more than one third of infected individuals may be reinfected. This result is in accordance with that obtained by Wearing & Rohani [27], who did not consider, as we do here, the possibility of highly sensitive boosting.

**Table 1:** Maximum likelihood parameter estimates for the three models and model comparison statistics.

	basic reproductiveratio, *R*_0_	infectiousperiod, 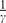 (wk)	immuneperiod, 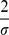 (yr)	boostingcoefficient, *κ*	notificationefficiency, *ρ*	AICweight	ΔAIC	LRT 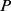 -value
SIRWS	16(12,19)	3.7(2.8,4.2)	34(17,66)	6.6(0.66,69)	0.15(0.15,0.16)	0.95	0	–
SIRS	18(16,19)	3.6(3.1,3.8)	192(178,192)	0	0.15(0.15,0.16)	0.05	6	0.012
SIR	17(16,17)	3.4(3.0,3.4)	∞	0	0.17	0.00	30	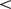 0.0001

Parameter estimates are given with 95% confidence intervals (in parentheses). We compare models using Akaike weights, 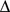
AIC values, and likelihood-ratio test (LRT) 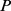-values, the latter being relative to the SIRWS model.

The maximum likelihood estimates (MLEs) of 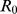 (16, CI:12–19) and the infectious period (3.7, CI:2.8–4.2 weeks) were clearly identified by these data (Fig. S3 in File S1), and are in agreement with previous estimates for pertussis from a variety of sources (e.g. [12], [22], [27]). The average duration of immunity induced by natural infection was estimated to be between 17 and 66 years. The seasonal transmission rate, 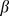, had a peak in December and a modest range of 3.8–4.8/wk (Fig. S4 in File S1).

### Large Cycles: Stochasticity and Waning Immunity

In simulations from both of the supported models (SIRS and SIRWS), even when not forced with the trends in birth and population size from Copenhagen, large cyclic outbreaks were often present (for example, Fig. 3). Secular trends in demography are therefore not a required ingredient for regime-switching behavior in this system.

**Figure 4:**
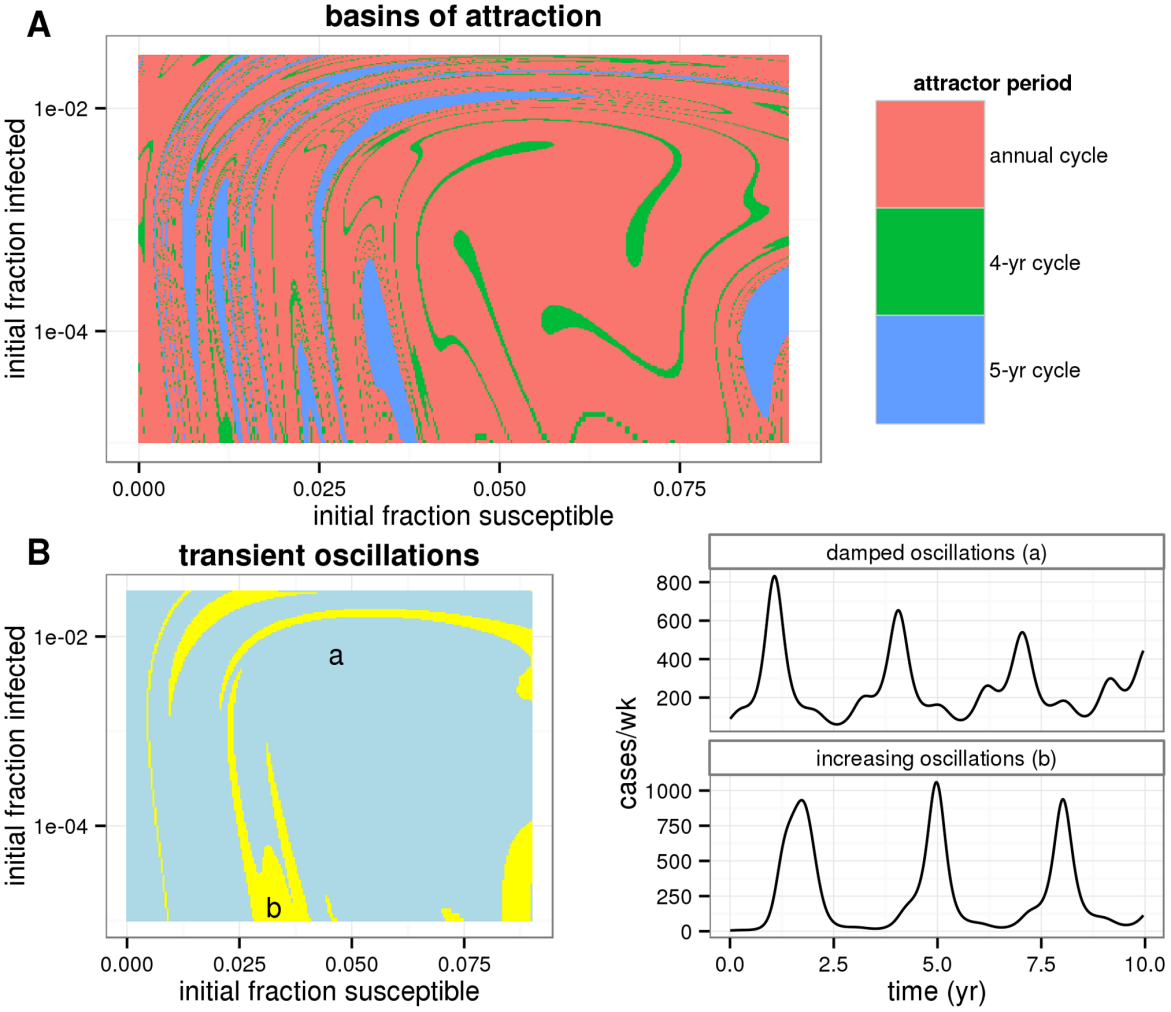
Basins of attraction and undamped transients. (A) The basins of attraction were computed using the maximum-likelihood estimates of parameters, but with a low birth rate of 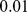/yr. Blue corresponds to a 5-yr cycle, green to a 4-yr cycle, and pink to the annual attractor. (B) Simulations from the same model and parameters but with a realistic birth rate of 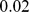/yr. Initial conditions that lead to oscillations with transiently increasing amplitude are indicated in yellow; blue indicates oscillations of steadily decreasing amplitude. Two example time series (from points labeled `a’ and `b’ are shown at right.

We therefore turn to the other two potential explanations of large cycles: stochasticity and nonlinearity. The SIRS and SIRWS models provide distinct explanations for the regime-switching behavior. The deterministic skeleton of the SIRS model offers only damped oscillations to a low-amplitude annual cycle. Stochastically forced, the model can produce episodes of large-amplitude cycles. The deterministic skeleton of the SIRWS model, by contrast, produces transient bursts of cycles that do not strictly decay in magnitude (Fig. 4b). In this model, immune waning and boosting create a positive feedback between incidence and population-level immunity. During periods of low incidence, boosting is infrequent and waning individuals lose protection (red line in Fig. 3, top). The resulting build-up of susceptibles provides fuel for large outbreaks. In these outbreaks, many individuals whose immunity has begun to wane (those in the W class) have their immunity boosted so that, at the end of the outbreak, most are in the fully protected class (R), and the stage is set for the cycle to repeat. In contrast, loss of immunity is a relatively constant process that is unrelated to incidence in the model without boosting (Fig. 3, bottom). The time spent in the large-amplitude regime increases with the boosting coefficient, 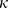, in the SIRWS model (Fig. S5 (left panel) in File S1), but also the degree of stochasticity, even in the absence of boosting (Fig. S5 (right panel) in File S1).

### Deterministic Dynamics: Attractors and their Ghosts

The deterministic skeleton of the SIRWS model in the vicinity of the MLE has a rich repertoire of dynamical behaviors. In regions of parameter space where birth rates are somewhat lower than they were in Copenhagen, one-, three- and four-yr cycles coexist with intermingled basins of attraction (Fig. 4a). In nearby parameter space, corresponding to Copenhagen’s historical birth rates, the only stable attractor is the smaller-amplitude annual cycle, yet certain initial conditions lead deterministically to transient bursts of large multi-annual outbreaks (Fig. 4b). These intermittent behaviors generates the transient multiannual cycles and represents the influence of the “ghost” of the nearby large-amplitude attractor.

### Model Predictions: Age-stratified Incidence and Vaccination

We hypothesized that the different mechanisms of immunity underlying the two best supported models would result in qualitatively different age distributions of cases, particularly during the high-amplitude cycles. The SIRWS model predicts that, during the deep troughs of this regime, loss of immunity is common, which leads to an increase in the number of re-infections, and therefore a higher age of infection, in this regime relative to the low-amplitude regime (Fig. S6 in File S1). Under the SIRS model, by contrast, the proportions of primary and repeat infections remain roughly constant. The Copenhagen data do indeed contain a disproportionately large number of cases in individuals over the age of 15 during the high amplitude period (chi-squared test for independence, 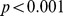, and Fig. S7 in File S1). However, stochastic simulations that track age from both models both predict a shift to cases in older individuals; during violently fluctuating periods the age of infection increases even in the model without boosting since there are long periods over which the force of infection is low (during the deeper troughs).

With mass vaccination there are much more pronounced differences between the models’ predictions, since vaccination effectively lowers the birth rate and thereby promotes large excursions under the boosting model (Fig. 3), with consequences for the age-specific incidence. The boosting model predicts more total cases (Fig. S8 (left), in File S1), larger amplitude outbreaks (Fig. S8 (right) in File S1) and more frequent outbreaks (Fig. S9 in File S1), with mean interepidemic period of 6 yr, as compared to 8 yr for the model without boosting. Additionally, both models predict an increase in cases in older individuals and a decrease in younger ones with the onset of vaccination. However, there are quantitative differences in the age distributions predicted by each model (Fig. S10 in File S1). Interestingly, neither model predicts the “honeymoon period” of low incidence observed in many highly vaccinated regions for the first few decades after the introduction of vaccination. This remains an enigma of whooping cough epidemiology.

## Discussion

We propose that spatial and temporal variability in pertussis cyclicity can be explained by a combination of stochasticity and nonlinear immunodynamics in which both waning and boosting are important. Under this model, stochasticity excites transient multiannual cycles of two types: damped oscillations around an annual attractor, on which population-level immunity fluctuates little, and the much more violently cyclic dynamics associated with large fluctuations in population-level immunity. We suggest that the mildly cyclic regions of the Copenhagen time series are an example of the former and that the three large peaks 1915–1923 were the result of a stochastic excitation of large-amplitude transients.

There are a number of biological realities not included in our model but potentially of great importance. First, we assumed homogeneous mixing between all age groups. Recent work has shown, however, that heterogeneities in mixing are detectible and important in pertussis dynamics [28]. Moreover, different age groups display different seasonalities in incidence testifying to age-stratified chains of transmission [16], [17], [29]. Second, immigration may have had a significant impact. The urbanization trend of the 20th century was partly responsible for the near-doubling of Copenhagen’s population over the period in question. We assumed that immigrants had the same profile of immunity as the resident population. However, rural areas may have seen less pertussis than urban regions, so it is conceivable that the immigrant population may have held a greater proportion of susceptible individuals. It is also worth noting that these data span World War I and the 1918 global flu pandemic. However, neutral Denmark actually saw increased life expectancy during the war and no large fluctuations in death rates [30]. Third, our model’s description of immunity using just two categories–fully immune (R) and waning (W)–is a simplification. A more elaborate model would posit a continuum of immune status, with associated variable susceptibilities, transmission rates, and reporting. Incorporating more immune classes than the two we consider would reduce the variance in the duration of immunity, a consideration which has been shown to promote cyclic dynamics. However, this seems unlikely to be driving the observed cyclicity; for this mechanism to generate limit cycles, 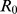 must be very low and the duration of immunity of the same order as the inter-epidemic period [31] in contradiction of observations [32]. Fourth, we have neglected the possibility of ecological interference between for example pertussis and measles, which has been demonstrated in other settings [33], [34]. However, over the period in question, measles and pertussis case reports are weakly but significantly *positively* correlated, not negatively correlated as one would expect were interference playing a large role (Fig. S11 in File S1). Nor is the 1914–1924 “regime shift” in pertussis mirrored in measles (Fig. S12 in File S1). Finally, we note that, while no long-term trends are visually evident in the data, it is possible that important parameters, such as reporting and transmissison rates, experienced trends in Copenhagen over this period. Our assumptions of constant reporting rate and periodic transmission rate are parsimonious, but we can not rule out the possibility of more complex time-dependence of these parameters. More generally, we note that, while the model with boosting is selected as the best explanation of the data from among the three candidates, no model-selection study can exclude the possibility that another, as yet unformulated, model might possess even greater explanatory power.

Our results suggest that the kinetics of immune waning and sensitivity to boosting may be important determinants of pertussis dynamics. If so, this would have significant implications for the efficacy of vaccination campaigns, since high levels of vaccination might lead to low incidence, infrequent immune boosting, and concomitant rapid waning of immunity [20]. Susceptible hosts might therefore accumulate in regions with little or no pertussis, increasing the potential for large outbreaks when the pathogen is once again locally re-introduced. Our results also highlight the need for a careful consideration of the changes that occurred with the introduction of vaccination. Puzzlingly, none of the models predicts the honeymoon period, an observation which requires us to posit alternative hypotheses for the initial impact of vaccination. Possible explanations may include heterogeneous mixing with respect to age, a slow increase in vaccine uptake, or vaccine-driven evolution. Plug-and-play statistical methods, such as the iterated filtering algorithm used here, make rigorous likelihood-based comparison of models practical in the face of stochastic and partially observed data, and thus make possible efficient extraction of what information pertinent to these issues is latent in time-series data. Our analysis further highlights some remaining important questions about pertussis epidemiology, in particular, the role of heterogeneous mixing and the consequences for transmission of repeat infections have yet to be fully elucidated. We were further able to identify specific model predictions–regarding age-distribution of cases and vaccine-era dynamics–that distinguish the two leading models and that can be tested using vaccine-era data. Looking ahead, it is clear that new information from vaccine-era dynamical data will be accompanied by new challenges to do with age-specific reporting bias, differences between natural and vaccine-induced immunity, and pathogen evolution. We anticipate, however, that model-based analyses that integrate genetic and serological data with age-specific incidence data and extract information from the disease’s dynamics will be especially valuable in resolving pertussis’ persistent puzzles.

## Methods

### The Data

Weekly case notifications from 1900 to 1937 were recorded and maintained by the municipality of Copenhagen (see [22] for details). City censuses were taken approximately every 5 yr and we estimated the population size at each week using a smoothing spline (with 10 df) on these data (Fig. S2 in File S1). The population grew from approximately 390,000 to 680,000 people during the study period. The model was stochastically forced with weekly birth data. The number of births per week showed no monotonic long-term trend but did vary, ranging from 124 to 325, with a mean of 212 and a standard deviation of 29 (Fig. S1 in File S1). We smoothed the birth data using a smoothing spline (with 10 df) and drew the number of births each week randomly from a Poisson distribution with the mean equal to the smoothed births for that week. During this time period, there was significant population growth of an average of 1.5% per yr. This was in part due to higher birth than death rates (0.0217 vs. 0.0135/person/yr respectively), accounting for about half of the population growth rate. The other 0.7%/year is attributable to an influx of domestic migrants to the city [22]. We assumed that the immune status of migrants entering the population mirrors that of the city residents and the death rate was independent of immune status. Therefore, in the model formulation the immigration rate (0.0068/person/yr) was subtracted from the death rate (0.0135/person/yr) to yield a total exit rate from the population, 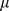, of 0.0067/person/yr.

### Time Series Analysis

Morlet’s wavelet transform of the square-root transformed weekly case notifications was computed [35], [36]. The signal-to-noise ratio for each week was calculated as the sum of the power in the three to four year period divided by the power in the less-than-half-year period. The crazy-climber ridge-finding method [35] was used to identify time periods of strong multiannual cyclicity both in the data and in simulations.

### The Models

The core of the model consists of four stochastic difference equations giving the weekly changes in the composition of the host population, one each for classes of people who are susceptible (S), infected and infectious (I), recovered and fully immune (R), and immune but susceptible to boosting (W). Susceptible recruitment occurs through births (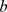), which are forced with weekly data, and loss of immunity, which occurs at a competing rate 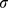. This rate competes with the boosting process, which occurs at a rate proportional to the boosting coefficient, 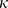 and the seasonally forced and environmentally stochastic force of infection, 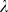. Individuals become infected at rate 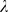 and recover at rate 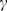. The mean infectious period is therefore 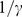. Recovered hosts begin to lose their immunity, moving from class 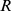 to 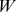 at rate 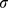, at which point they may either fully lose their immunity or be boosted. In the absence of boosting, the average duration of immunity is 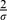. Death and migration are accounted for in the weekly survival probability, 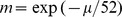 where 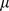 is the per capita annual exit rate, described above. The models are nested: when 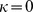, the model collapses to the SIRS model and the SIR model is obtained by setting 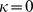 and 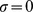. Table S1 in File S1 summarizes the model parameters and their meanings. The model is diagrammed in Fig. 2; its mathematical representation is:
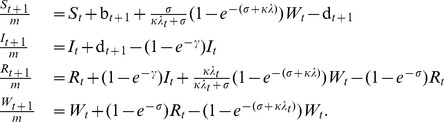



Stochasticity is incorporated via births and the frequency-dependent force of infection according to:
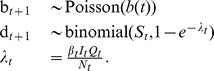



As discussed above, the expected number of births 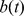 is taken from smoothed demographic data. Infections follow a binomial process in which the probability of success varies according to a seasonally fluctuating 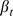 and environmentally stochastic 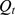. The seasonality in transmission is incorporated via three periodic cubic spline bases. Each basis function has a period of 52 weeks and mean value 1; they are offset by 17–1/3 weeks and weighted by 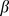, a vector of 3 coefficients. 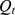 is temporally uncorrelated, multiplicative white noise with unit mean and standard deviation 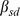. To obtain these features, it is convenient to use a gamma distribution:
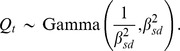



In order to fruitfully compare the model and data, we must further contend with under-reporting. The notification efficiency, or probability of an infection being reported, has been shown to be quite low for pertussis, falling below 10% in the current era and estimated at 10 to 20% in the prevaccine era [37], [38]. Additionally, overdispersion is ubiquitous and failure to account for it can lead to spurious conclusions [39]. We therefore define a model by which reported cases are related to true incidence according to an over-dispersed binomial distribution,
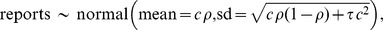
where 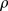 is the notification efficiency, and 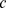 the number of cases reported. This process has greater variance than a standard binomial process with mean 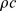 and standard deviation 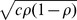 when 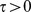, determined by 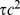. The degree of overdispersion is proportional to the number of reported cases, in accord with observations that some doctors tended to report more cases during the peak of an epidemic [22], [40].

### Parameter Estimation

We estimated twelve parameters for the full model: three seasonal transmission coefficients, 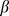, 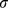, 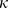, 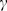, 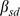, 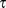, 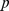, and three initial conditions (four state variables less one constraint). One thousand starting parameter combinations were chosen using a latin hypercube. The initial conditions for each set of starting parameters was optimized using trajectory matching followed by probe matching [41] using synthetic likelihood [42]. The probes used were third-degree polynomial regression, the autocorrelation function at lags of 0, 1, 2, 3, 4, 26, 52, 78, and 104 wk and the mean and median numbers of reports. The parameter combinations with the forty best synthetic likelihoods were used to start the iterated filtering procedure based on the full likelihood, computed by sequential Monte Carlo [43]. This procedure was performed on the best points repeatedly. New starting points were chosen to fill in gaps in the likelihood surface by interpolating along ridges in likelihood space. For example, the seasonality was strongly identified, i.e. the ratios among the three 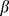
s showed little variability. We therefore were able to choose starting values with relatively high likelihoods for transmission rates (mean 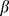) and 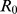 that we had not yet explored by shifting the 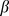
s with respect to each other. We also did this for 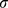 and 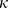, which also have a well-defined relationship. Starting parameters for the model without boosting (SIRS) and without waning (SIR) were selected from the likelihood surface of the full model near 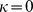 and 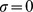 respectively. All estimation procedures were carried out using the package pomp [25] for the R statistical computing environment [44].

### Simulations

Summary statistics of stochastic simulations were computed from 100 simulations at each parameter combination listed in legends to Figs. S5 and S7 in File S1. The boosting coefficient 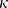 and the environmental stochasticity 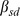 were varied. For the rapid waning simulations all other parameters were fixed at the maximum likelihood estimates of the full model. For the slow-waning simulations, the parameters with the highest likelihood given that 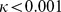 were used. Vaccination was implemented by moving 90% of births directly to the recovered class, corresponding to the assumption of 90% effective coverage. Pearson’s chi-squared test for independence was used to assess differences in simulated age distributions from the SIRS and SIRWS models in the vaccine era.

The basins of attraction for the SIRWS model (Fig. 4a) were identified through simulation from the deterministic skeleton of the stochastic model at 10,000 starting points, using the maximum likelihood estimates for all parameters, the birth rate fixed at 0.01/yr. In addition to removing stochasticity, the birth and death rates were taken to be equal so the model could reach a stable attractor. The presence of transient oscillations of increasing amplitude was identified for the same initial conditions and parameters except the birth rate was fixed at 0.02/yr, which is within the range of actual birth rates in Copenhagen at the beginning of the 20th century.

## Supporting Information

File S1
**Supporting information, including Figures S1–S12 and Table S1.** Table S1, Symbols appearing in the model equations, with their interpretations. Figure S1, Births in Copenhagen. Figure S2, Copenhagen population size. Figure S3, Likelihood profiles for estimated parameters. Figure S4, Estimated seasonality of transmission. Figure S5, Effect of 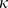 and 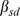 on the time spent in multiannual cycles. Figure S6, Proportion of post-primary cases during cyclic and acyclic regimes. Figure S7, Proportion of cases in people over the age of 15 years. Figure S8, Distributions of total annual and peak incidence. Figure S9, Periodicity of simulated epidemics. Figure S10, Annual age-stratified incidence in simulated epidemics. Figure S11, Lack of correlation between measles and pertussis cases. Figure S12, Time series of measles and pertussis in Copenhagen.(PDF)
