# Correction: Immune Boosting Explains Regime-Shifts in Prevaccine-Era Pertussis Dynamics

**DOI:** 10.1371/annotation/5564cacb-8694-433f-991e-e1cc29a4f4bf

**Published:** 2014-01-29

**Authors:** Jennie S. Lavine, Aaron A. King, Viggo Andreasen, Ottar N. Bjørnstad

Errors occurred in the preparation of Figure S3. Please see the corrected Figure S3 here: 
