# Supplementary material for: Immune Boosting Explains Regime-Shifts in Prevaccine-Era Pertussis Dynamics
Source: PLoS One. 2013 Aug 26;8(8):e72086. doi: 10.1371/journal.pone.0072086 (PMC3753349; doi:10.1371/journal.pone.0072086)
Supplement: File S1 — Supporting information, including Figures S1–S12 and Table S1. Table S1, Symbols appearing in the model equations, with their interpretations. Figure S1, Births in Copenhagen. Figure S2, Copenhagen population size. Figure S3, Likelihood profiles for estimated parameters. Figure S4, Estimated seasonality of transmission. Figure S5, Effect of and on the time spent in multiannual cycles. Figure S6, Proportion of post-primary cases during cyclic and acyclic regimes. Figure S7, Proportion of cases in people over the age of 15 years. Figure S8, Distributions of total annual and peak incidence. Figure S9, Periodicity of simulated epidemics. Figure S10, Annual age-stratified incidence in simulated epidemics. Figure S11, Lack of correlation between measles and pertussis cases. Figure S12, Time series of measles and pertussis in Copenhagen. (PDF) [file pone.0072086.s001.pdf]

Immune boosting explains regime-shifts in prevaccine-era pertussis dynamics: Supporting Information

Jennie S. Lavine, Aaron A. King, Viggo Andreasen, Ottar N. Bjørnstad

Supplementary Table

Table S1. Symbols appearing in the model equations, with their interpretations. Also shown are their units and whether their values were estimated or taken directly from data.

symbol	interpretation	units	estimated or data?
σ	rate of loss of immunity	1/wk	estimated
$\boldsymbol{\beta}$	seasonal transmission rates	1/wk	estimated
κ	boosting coefficient	–	estimated
γ	recovery rate	1/wk	estimated
β_{sd}	environmental stochasticity	–	estimated
τ	overdispersion in reporting	–	estimated
ρ	observation probability	–	estimated
c	reported cases	–	data
b	births	–	data

Supplementary Figures

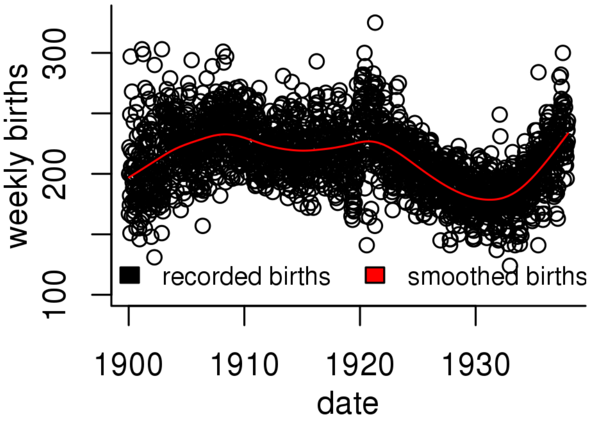

Figure S1. Births in Copenhagen. The black dots show the recorded number of weekly births; the red line the smoothed births, which were used as the mean of Poisson draws for weekly births in the model.

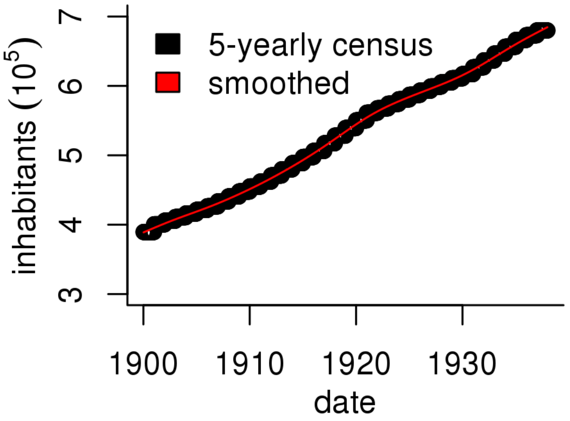

Figure S2. Copenhagen population size. The black dots show the censused population size, taken every five years. The red line shows the smoothed population size with which the model was forced.

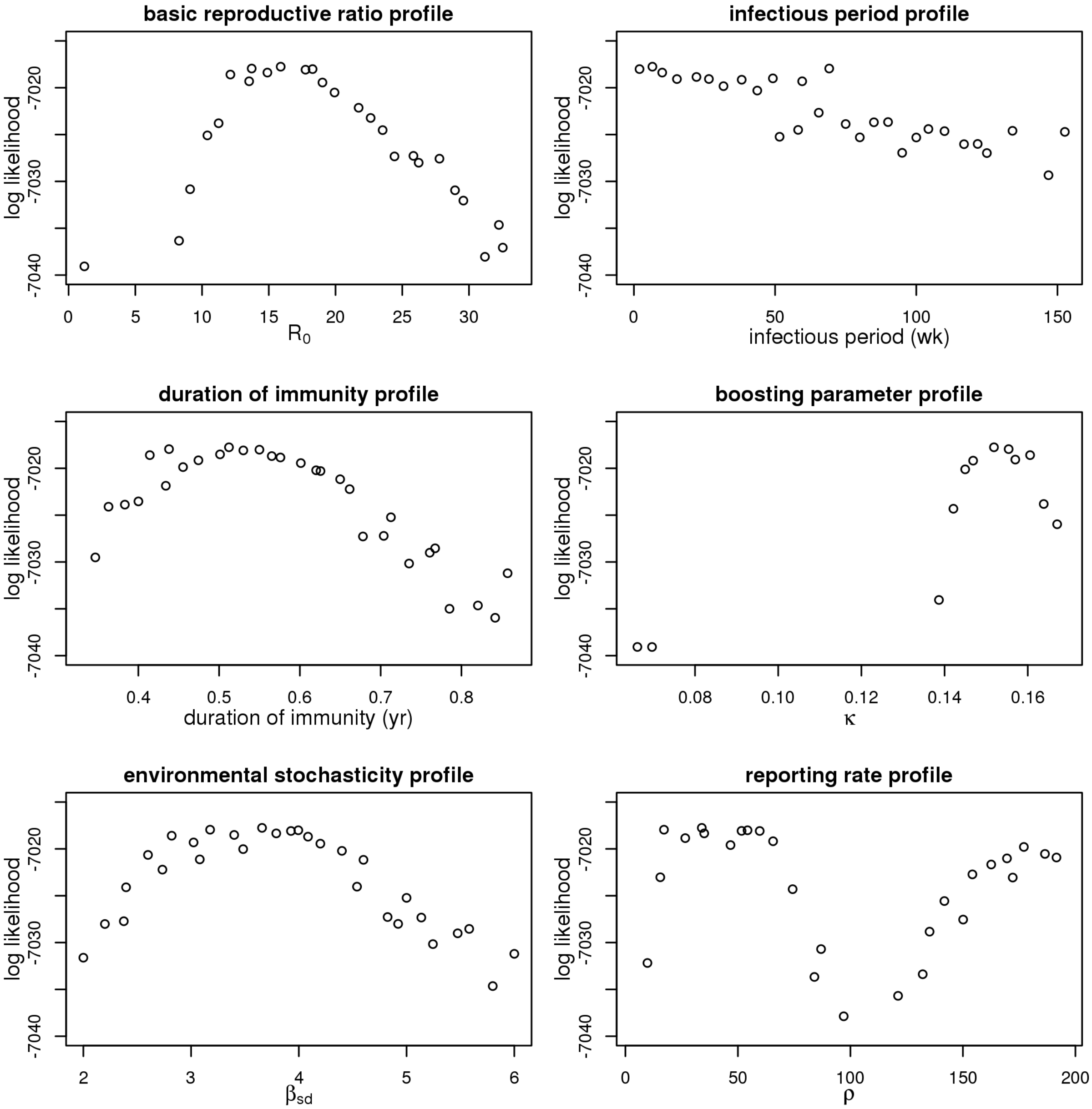

Figure S3. Likelihood profiles for estimated parameters.

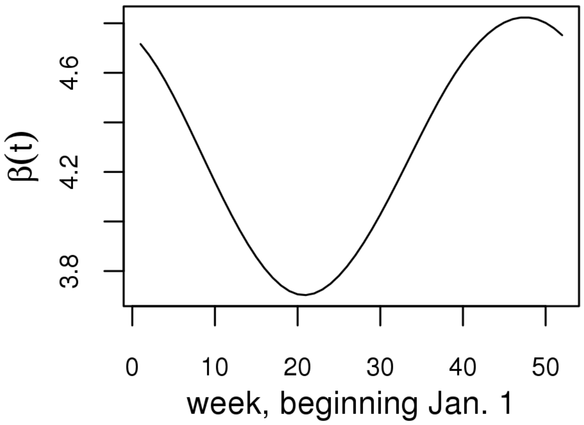

Figure S4. Estimated seasonality, computed from the estimates of the three βs and the B-spline basis functions.

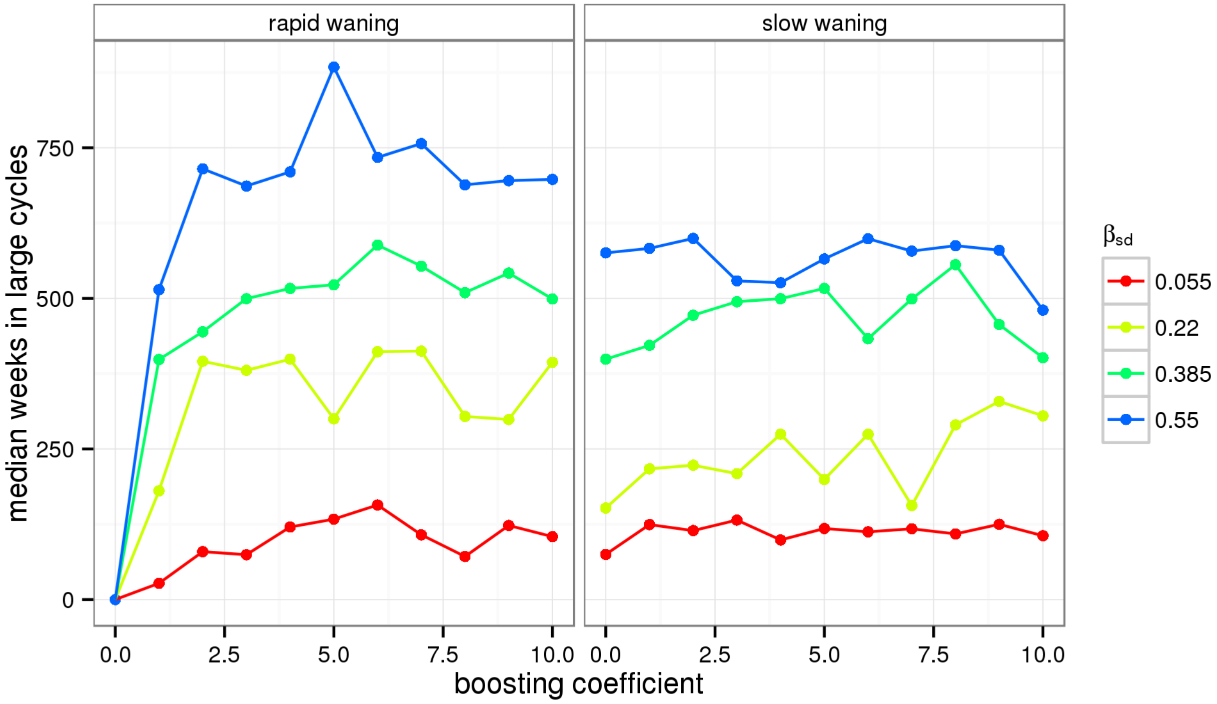

Figure S5. Effect of κ and β_{sd} on the time spent in multiannual cycles. Parameters used for rapid waning simulations: $\beta_1 = 5.0$, $\beta_2 = 3.2$, $\beta_3 = 4.8$, $\gamma = 0.27$, $\sigma = 1.1\times10^{-3}$, $\tau = 7.5\times10^{-4}$, $\mu = 1.3\times10^{-4}$, $\rho = 0.15$, $S_0 = 0.049$, $I_0 = 7.7\times10^{-4}$, $R_0 = 0.95$, $W_0 = 2.9\times10^{-3}$. Parameters used for slow loss of immunity simulations: $\beta_1 = 5.2$, $\beta_2 = 3.4$, $\beta_3 = 5.0$, $\gamma = 0.24$, $\sigma = 2.2\times10^{-4}$, $\tau = 7.7\times10^{-4}$, $\mu = 1.3\times10^{-4}$, $\rho = 0.15$, $S_0 = 0.043$, $I_0 = 8.7\times10^{-4}$, $R_0 = 0.95$, $W_0 = 0.0029$

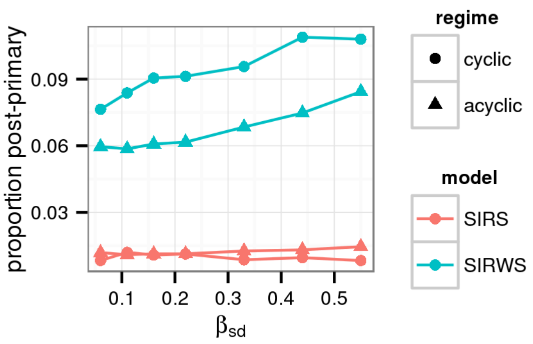

Figure S6. Effects of stochasticity on the proportion of cases that are post-primary during the cyclic regime (circles) and acyclic regime (triangles) for stochastic models with rapid (pink lines) vs slow (blue lines)loss of immunity, and the boosting coefficient, $\kappa = 7$. Parameters used for rapid waning simulations: $\beta_1 = 5.0$, $\beta_2 = 3.2$, $\beta_3 = 4.8$, $\gamma = 0.27$, $\sigma = 1.1{\times}10^{-3}$, $\tau = 7.5{\times}10^{-4}$, $\mu = 1.3{\times}10^{-4}$, $\rho = 0.15$, $S_0 = 0.049$, $I_0 = 7.7{\times}10^{-4}$, $R_0 = 0.95$, $W_0 = 2.9{\times}10^{-3}$. Parameters used for slow loss of immunity simulations: $\beta_1 = 5.2$, $\beta_2 = 3.4$, $\beta_3 = 5.0$, $\gamma = 0.24$, $\sigma = 2.2{\times}10^{-4}$, $\tau = 7.7{\times}10^{-4}$, $\mu = 1.3{\times}10^{-4}$, $\rho = 0.15$, $S_0 = 0.043$, $I_0 = 8.7{\times}10^{-4}$, $R_0 = 0.95$, $W_0 = 0.0029$

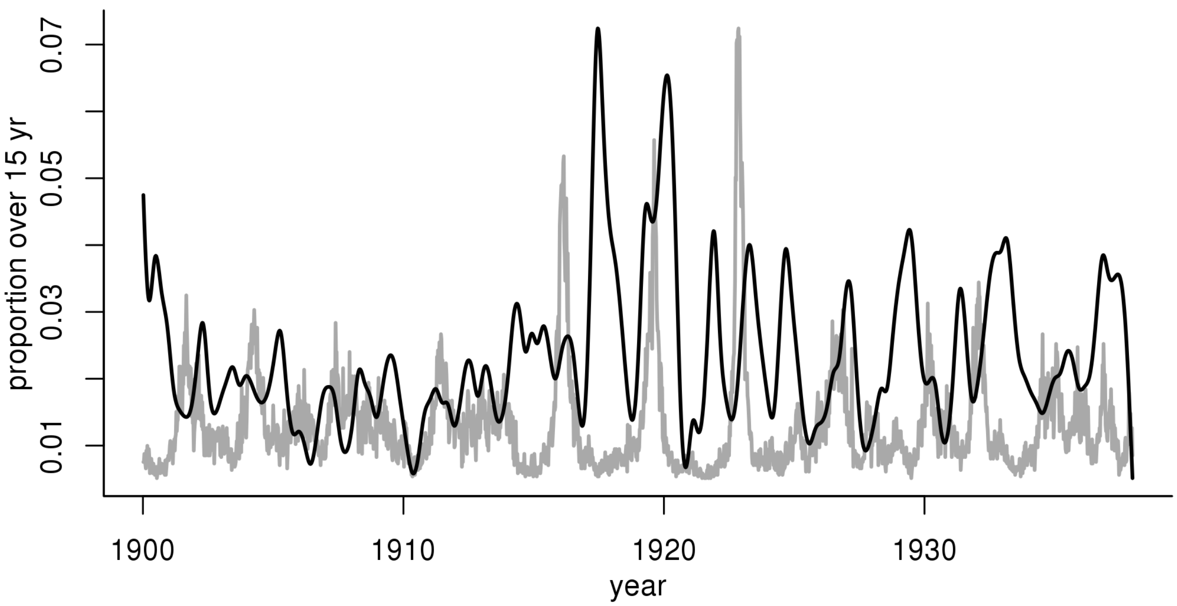

Figure S7. The black line shows the smoothed proportion of cases in people over the age of 15 years. The gray line, which shows the fluctuation in incidence as shown in figure 1 of the main text, is included to visually highlight the relationship between the highly cyclic regime and the increased proportion of cases in people over the age of 15 years.

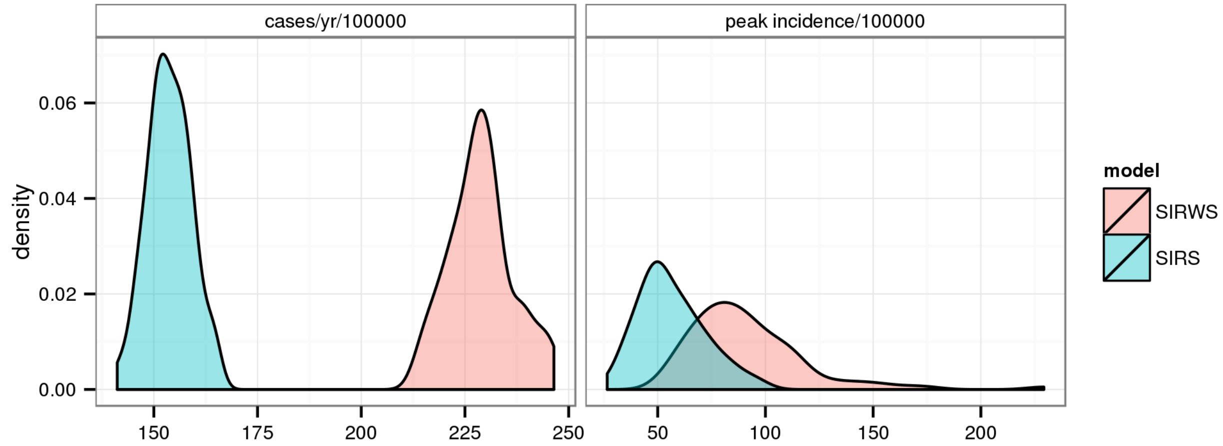

Figure S8. Kernel density estimates of distributions of (a) total annual incidence per 100,000 and (b) peak case reports per week per 100,000 in the vaccine era. Calculated from same simulation data as Fig. S9.

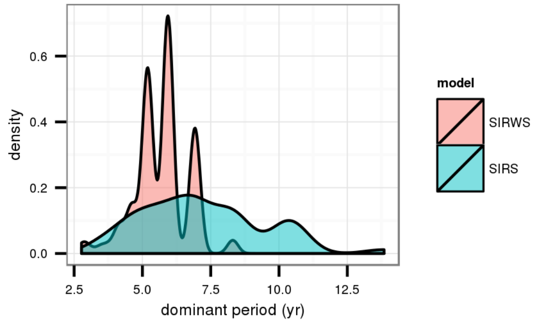

Figure S9. Distribution of dominant periods in 100 simulations from each model, calculated from 80 yr of simulations, starting 20 yr after the onset of vaccination.

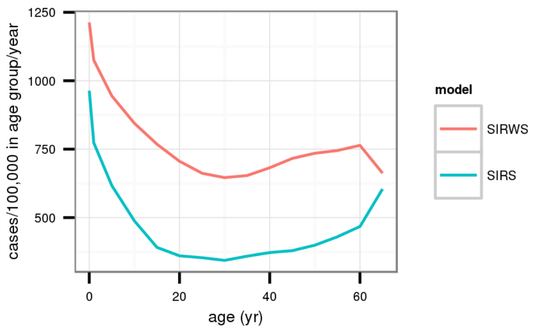

Figure S10. Annual incidence per 100,000 people in each age group, calculated from same simulations as figure S9

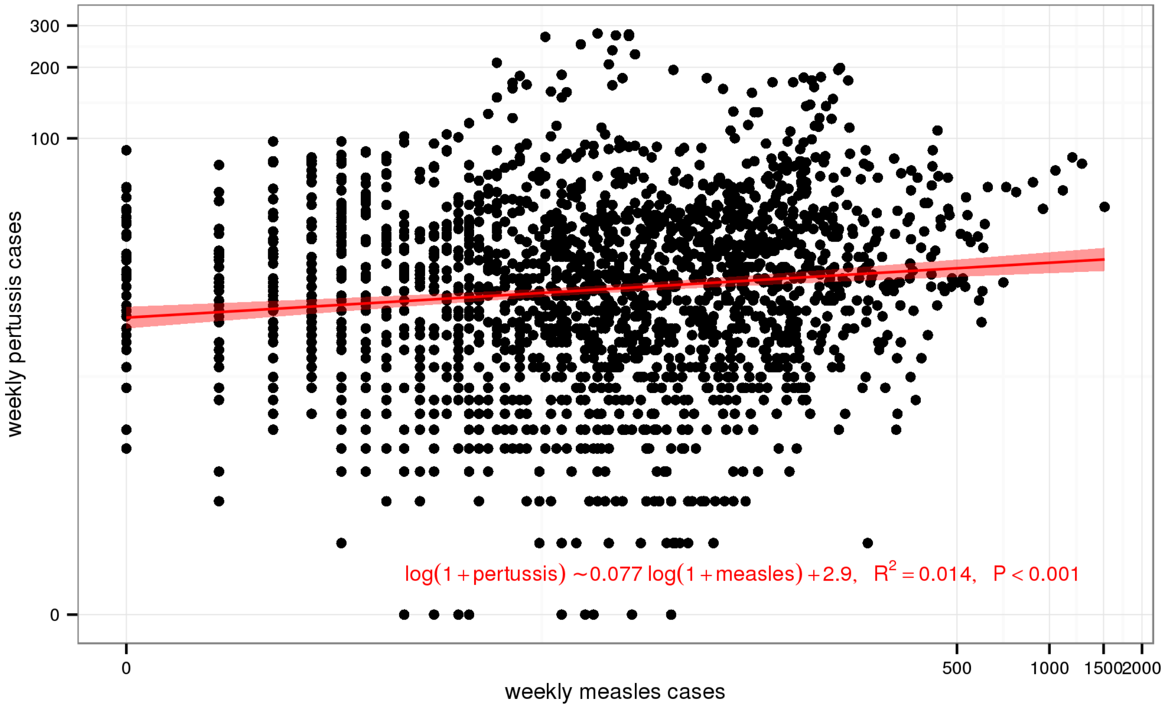

Figure S11. The correlation between measles and pertussis cases is weak and statistically significantly positive. Were ecological interference occurring between these infections, one might expect to see a negative correlation [1].

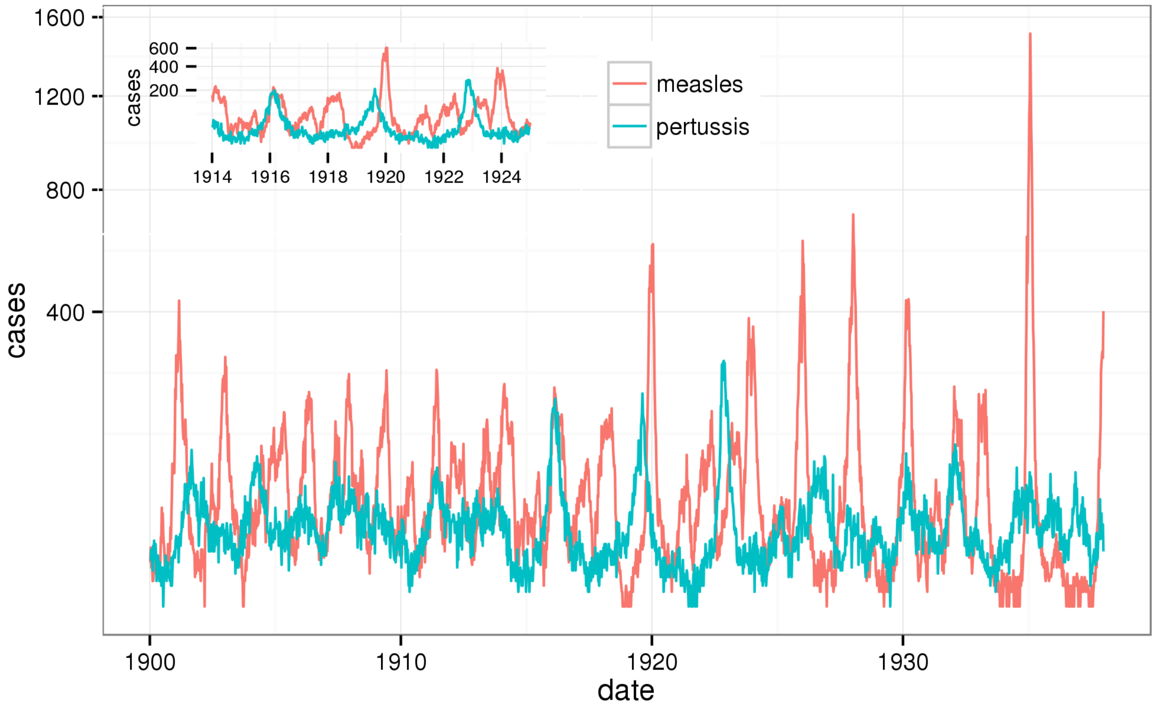

Figure S12. Time series of measles and pertussis in Copenhagen. The inset shows a detail of the 1914–1924 period. No clear pattern indicative of ecological interference between these infections is evident.

Supplementary Reference

1. Rohani P, Earn DJ, Finkenstdt B, Grenfell BT (1998) Population dynamic interference among childhood diseases. Proc R Soc Lond B 265: 2033–2041.
