# Supplementary figures and images for: Correction: Immune Boosting Explains Regime-Shifts in Prevaccine-Era Pertussis Dynamics
Source: PLoS One. 2014 Jan 29;9(1):10.1371/annotation/5564cacb-8694-433f-991e-e1cc29a4f4bf. doi: 10.1371/annotation/5564cacb-8694-433f-991e-e1cc29a4f4bf (PMC3905976; doi:10.1371/annotation/5564cacb-8694-433f-991e-e1cc29a4f4bf)

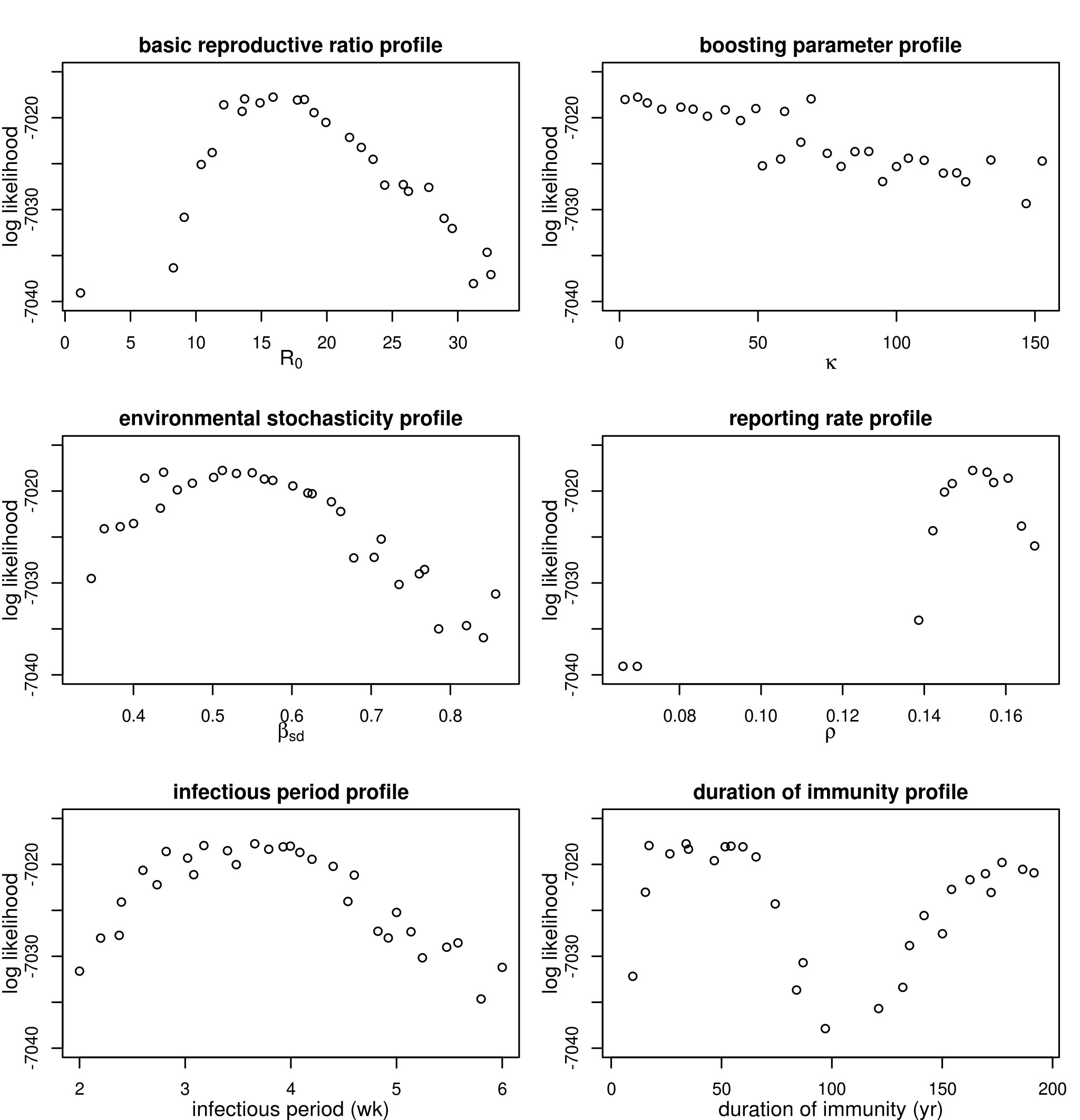

Supplement: Supplementary file 1 [file pone.5564cacb-8694-433f-991e-e1cc29a4f4bf.s001.tif]
